# Causes of burnout syndrome and coping strategies among high dependency unit nurses of an institution in the greater Accra region of Ghana

**DOI:** 10.1002/nop2.1052

**Published:** 2021-09-01

**Authors:** Yaa Adomaah Boateng, Sylvia Ama Osei, Irene Korkoi Aboh, Andrew Adyei Druye

**Affiliations:** ^1^ School of Peri Operative and Critical Care Nursing Korle‐Bu Teaching Hospital Accra Ghana; ^2^ School of Nursing and Midwifery University of Cape Coast Cape Coast Ghana

**Keywords:** burnout, coping strategies, high dependency unit, nurse

## Abstract

**Aim:**

The aim of this study was to determine the causes of burnout syndrome and identify strategies to cope with them among nurses at high dependency unit if a regional Hospital in Ghana.

**Design:**

A descriptive convenient and purposive cross‐sectional study design was used for this study.

**Methods:**

The study involved 40 nurses in the high dependency unit of the Hospital. A Maslach Burnout Inventory (MBI) was adopted to measure burnout experienced by these nurses concentrating on feelings of being emotionally exhausted and lack of energy, depersonalization, feelings of impersonal response towards patients and personal achievement.

**Results:**

62.5% of the respondents were in the high emotional exhaustion, 55% scored high in the depersonalization section and 52.5% scored high in the personal achievement (competence) on the Maslach Burnout Inventory (MBI).

## 1 BACKGROUND

Social changes over the last decades have led to changes at the workplace. This has led to physical and emotional exhaustion among workers resulting in excessive accumulation of workload and high burnout levels (Paiva et al., 2016). High burnout directly affects the physiological stability of individuals pre‐disposing them to cardiovascular diseases, psychiatric disorders and gastrointestinal disorders (Paiva et al., 2016). The term “burnout” was coined by Freudenberger in 1974 to describe workers' reactions to the chronic stress commonly found in occupations involving numerous interpersonal interactions (Doulougeri et al., 2016). The document added that burnout is a psychological syndrome that involves a prolonged response to chronic emotional and interpersonal stressors. In addition, burnout syndrome refers to a situation in which workers appear disconnected from their job and everything seems to be senseless (Lorenz et al., 2010). Literally, this implies that the worker loses the sense of relationship to work.

Burnout is a syndrome comprises of three dimensions; the first of which is emotional exhaustion, which is when someone is “emotionally overextended and exhausted by work.” The second is depersonalization, which is categorised by “unfeeling and impersonal response towards recipients of one's care or service” and lastly low personal achievement, which is when people have “feelings of competence and successful achievement with people,” which results in negative work experiences (Doulougeri et al., 2016). It is a common problem among healthcare professionals, especially those at the frontline of healthcare delivery. Burnout affects nearly half of all nurses and physicians in developed countries, and it is associated with poor outcomes such as worse patient safety (Dolan et al., 2015).

In healthcare, burnout contributes to poor outcomes, including worse patient safety and lower patient satisfaction (Cimiotti et al., 2012). Burned‐out employees are more probably to leave their jobs, take sick leave and suffer from depression and relationship problems. In addition, burnout can result in work‐related consequences such as dissatisfaction with the work, reduction in the quality of care, unjustified absenteeism, intention of giving up their job and abandonment (Dyrbye et al., 2011).

Burnout is a complex phenomenon because it is not manifested in one single form and is influenced by broad social, cultural and professional factors. Different burnout measurement tools such as Professional Quality of Life Scale, Copenhagen Burnout Inventory and Maslach Burnout Inventory is used to measure occupational burnout. However, the Maslach Burnout Inventory (MBI) is the most commonly used measure of burnout. The MBI describes burnout according to three components (emotional exhaustion, depersonalization and reduced personal accomplishment) and provides the opportunity to assign a classification of burnout into low, medium or high burnout (Doulougeri et al., 2016).

Identifying and characterizing burnout is important as it can have a negative impact on providers and patient care. To help prevent burnout among health professionals and promote patient safety, there is a need to explore the causes and strategies to cope with burnout among nurses.

## 2 AIM

The aim of this study was to determine the causes of burnout syndrome and identify strategies to cope with them among nurses at high dependency unit if a regional Hospital in Ghana.

## 3 METHODS AND MATERIALS

A descriptive cross‐sectional study was used to determine the causes of burnout syndrome and identify strategies to cope with them among nurses at a Regional Hospital over a study period of 3 months. The study was conducted among high dependency unit nurses in one of the Regional Hospitals in Ghana. The hospital has an ultra‐modern facility of about 620 beds, with the full complement of specialist services that would adequately meet the growing demand for quality secondary and tertiary level healthcare services in Ghana (Laryea, 2017). It has seven (7) major departments namely Accident and Emergency Department, Surgery Department, Medical Department, Paediatrics Department, Obstetrics and Gynaecology Department, Theater and Allied Health Department. Each department has units and subunits. The hospital has HDU, and all nurses in the units were enlisted to take part in the study. The dependency unit consists of intensive care unit (ICU), recovery ward and emergency unit of the Hospital (Egungwu, 2015).

### 3.1 Population

The study was conducted among the high dependency unit nurses working at intensive care unit (ICU), recovery ward and emergency unit of the Hospital. Inclusion criteria was all permanent nurses working at the Hospital's DHU and those who are ready to participate in the study. The exclusion criteria are the other recognised staff such as doctors, pharmacists, anaesthetists and other paramedics.

### 3.2 Sample size and sampling procedure

Nurses who qualify for this study were 45; a convenient and purposive sampling technique was used for the study. This non‐probability sampling was decided because the number of nurses in these units was 45, and so researchers decided to use all for the sake of precision.

### 3.3 Data collection

Structured questionnaires based on research objectives were developed for the purpose of data collection after reviewing relevant literature. Data were collected by the principal investigator. The questionnaires used solicited information on socio‐demographic data, the proportion of nurses with burnout syndrome, causes of burnout syndrome and strategies of coping with burnout syndrome. A Maslach Burnout Inventory (MBI) was adopted to measure burnout experienced by these nurses. The MBI is the most widely used inventory to assess burnout, and it consists of 22 questions across three dimensions: emotional exhaustion (first nine questions); feelings of being emotionally exhausted and lack of energy, depersonalization (next five questions); feelings of impersonal response towards recipients of the service and personal achievement (last eight questions); feeling of competence. Each question is scored on a scale from 0 (never) to 6 (everyday). The points from each dimension are added to provide a total score for that dimension. The score for each dimension can be categorized as low, moderate or high: emotional exhaustion (low ≤13; moderate 14–26; high ≥27); depersonalization (low ≤5; moderate 6–9; high ≥10); and personal achievement (high ≤33; moderate 34–39; low ≥40). Higher scores on emotional exhaustion and depersonalization and a lower score on personal achievement are associated with higher burnout (Doulougeri et al., 2016).

### 3.4 Validity and reliability of questionnaire

To ensure validity and reliability, questionnaire was developed and structured guided by objectives of the study and information from the reviewed literature. The questionnaire was given to a supervisor who had a lot of experience in the area of study for input and the necessary corrections made. A pre‐test was carried out using 15 nurses at 37 military hospitals to check for consistency, relevance and applicability of the questionnaire. These 15 questionnaires were entered into the SPSS Version 25.0. Cronbach's alpha was ran for the 22 items on the Maslach Burnout Inventory (MBI) on a Likert scale, and the reliability coefficient was 0.712.

### 3.5 Data collection procedure

The establishment of good rapport with the nurses before the actual data collection at the study institution was ensured. Data were collected through one‐on‐one administration of questionnaires and retrieved in 1–2 weeks after administering them. Prior to the data collection, permission to carry out the research was obtained from the hospital. Study participants were not forced into the study rather through written and verbal informed consent. Respondents’ confidentiality was assured through the use of numbers to identify the questionnaires.

### 3.6 Ethical consideration

An introductory letter from the School of Perioperative and Critical Care Nursing was obtained and sent to the Research and Development unit of the Regional hospital where the study was conducted before the commencement of the study. Permission was also sought from the nurse manager at the hospital concerning the participation of her nurses. Respondents were informed that participation is voluntary, and any information given would be confidential. They were told not to write their names on the questionnaire to ensure anonymity.

### 3.7 Data analysis procedure

Collected data were managed in Microsoft Excel and analysed using SPSS version 25. Features of the data gathered were explored to get a general description of responses given by respondents. Descriptive statistics were used to present data on the population by using percentages in tables. A Pearson chi‐square test was done to determine factors associated with burnout syndrome at a confidence interval of *p*‐value <.05 a considered statistically significant level.

## 4 RESULTS

Study samples were described in terms of their socio‐demographic characteristics and the proportion of nurses with burnout syndrome. Then, causes of burnout syndrome and strategies of coping with burnout syndrome are analysed. Table 1 is the percentage distribution of the socio‐demographic characteristics of population used for the study. Forty‐five questionnaires were sent out, but only 40 came back creating an 89% return rate. Majority (67.9%) of the respondents were females with age range of 29–58 years. Half (50%) of the nurses had Bachelor's degree, 30% had Diploma certificate and the rest (7.5%) had master's degree and were critical care nurses (45%), Registered Nurses (35%) and 20% were Emergency Nurses. Most (47.5%) of the respondents had worked for a minimum of 6 years, 22.5% had worked for 3–5 years, 17.5% had worked for more than 10 years and 12.5% had less than 3 years working experience as nurses.

**TABLE 1:** Socio‐demographic characteristics of respondents

Variable	Frequency (*N* = 40)	Per cent (%)
Age group in years		
Less than 29	3	10.0
30–39	22	55.0
40–49	11	27.5
50–59	4	7.5
Gender		
Female	27	67.5
Male	13	32.5
Highest Educational Level/Qualification		
Diploma certificate	12	30.0
Bachelor's degree	25	62.5
Masters	3	7.5
Nursing Specialty		
Critical care nurse	18	45.0
Emergency nurse	8	20.0
Registered General nurse	14	35.0
Years of Work Experience		
Less than 3 years	5	12.5
3–5 years	9	22.5
6–10 years	19	47.5
More than 10 years	7	17.5

Note

Field data 2017.

### 4.1 Proportion of nurses with burnout syndrome

Table 2 shows the distribution of nurses by dimensions of the MBI according to the categories of low, moderate and high. More than half (62.5%) of the respondents were in the high emotional exhaustion (EE) range with a mean of 28.4, 55% scored high in the depersonalization (DP) section with a mean of 9.7 and 52.5% scored high in the personal achievement (PA) section with a mean of 33 on the scale. Thus, more than half of the respondents felt emotionally exhausted and lack of energy from their job, over half of them had impersonal response towards recipients of the service and a little above half of them were found to be highly competent in their job.

**TABLE 2:** Distribution of respondents by dimensions of the MBI

Dimension	Category	Range	*N* = 40	Per cent (%)
Emotional exhaustion (EE)	Low	≤13	7	17.5
	Moderate	14–26	8	20
	High	≥27	25	62.5
Depersonalization (DP)	Low	≤5	6	15
	Moderate	6–9	12	30
	High	≥10	22	55
Personal Achievement (PA)	Low	≥40	21	25
	Moderate	34–39	9	22.5
	High	≤33	10	52.5

Note

**Emotional Exhaustion: mean 28.4; standard deviation 12.4; median 28.

**Depersonalization: mean 9.7; standard deviation 3.6; median 10.

**Personal Achievement: mean 33; standard deviation 7; median 32.5.

### 4.2 Causes of burnout syndrome

A majority (77.5%) of respondents indicated poor conditions of work, work overload (67.5%), low wages (97.5%), emotionally upsetting situations (72.5%), handling a large number of patients alone (60%), lack of break during shift (65%) and inadequate nursing staff (62.5%) as causes of burnout that applied to them. On the other hand, a 70% of respondents indicated lack of recognition of the profession, doctor/nurse conflicts (62.5%) and too frequent night duties (62.5%) as being causes of burnout. Table 3 displays causes of burnout among respondents.

**TABLE 3:** Causes of burnout among respondents

Variable	Frequency (*N* = 40)	Per cent (%)
Poor conditions of work		
Yes	31	77.5
No	9	22.5
Work overload		
Yes	27	67.5
No	13	32.5
Low wages		
Yes	39	97.5
No	1	2.5
Emotionally upsetting situations		
Yes	29	72.5
No	11	27.5
Lack of recognition of the profession		
Yes	12	30.0
No	28	70.0
Doctor/Nurse conflicts		
Yes	15	37.5
No	25	62.5
Handling a large number of patients all alone		
Yes	24	60.0
No	16	40.0
Lack of break during shifts		
Yes	26	65.0
No	14	35.0
Too frequent night duties		
Yes	15	37.5
No	25	62.5
Inadequate nursing staff		
Yes	25	62.5
No	15	37.5
Others		
Bad attitude from colleagues	9	22.5
Inadequate resources to work with	11	27.5
Not being appreciated by In‐charges or Nurse managers	13	32.5
Nurse/managers conflicts	7	17.5

Note

Field data 2017.

Respondents further stated not being appreciated by their in charges or nurse managers (32.5%), inadequate resources to work with (27.5%), bad attitude of colleagues (22.5%) and nurse and managers conflicts (17.5%) cause burnout among them.

### 4.3 Strategies of coping with burnout syndrome

Table 4 depicts coping strategies that best helped to relieve burnout. Majority (42.5%) of the respondents indicated that problem‐focused coping strategy, emotional support from family/friends' strategy (22.5%), using humour as a coping strategy (15%), listening to music (12.5%) and emotion‐focused strategy (7.5%) best relieve burnout.

**TABLE 4:** Coping strategies that best helped to relieve burnout

Variable	Frequency (*N* = 40)	Per cent (%)
Emotion‐focused strategy	3	7.5
Listening to music	5	12.5
Obtaining emotional support from family/friends	9	22.5
Problem‐focused coping strategy	17	42.5
Using humour as a coping strategy	6	15.0

Note

Field Data 2017.

Table 5 displays measures that can be put in place to help nurses cope with burnout in their units. Majority (30%) of the respondents indicated adequate motivation of staff can help nurses cope with burnout in their units, 27.5% indicated that setting up hospital counselling units can help nurses cope with burnout in their units, 22.5% indicated providing adequate resources for nurses to work with can help nurses cope with burnout in their units, 12.5% indicated creating good working environment and good relationship with both staff and patients can help nurses cope with burnout in their units and 7.5% indicated increasing staff strength can help nurses cope with burnout in their units.

**TABLE 5:** Measures for coping with burnout

Variable	Frequency (*N* = 40)	Per cent (%)
Adequate motivation of staff	12	30.0
Creating good working environment/good relationship with staff and patients	5	12.5
Increase staff strength	3	7.5
Providing adequate resources for nurses to work with	9	22.5
Setting up Hospital counselling unit	11	27.5

Note

Field Data 2017.

## 5 DISCUSSION

For many years, conventional wisdom held that employees work primarily for money and could be motivated through financial reward and fear, a realization that performance at work place is much more complex (Banks, 1997). Higher scores on emotional exhaustion and depersonalization and a lower score on personal achievements are associated with higher burnout (Doulougeri et al., 2016). This study revealed that more than half of the respondents felt emotionally exhausted and a lack of energy from their job and characteristic, some had impersonal response towards recipients of the service (depersonalization) and a few were found competent in their job (personal achievement). This implies that despite this, more than half of them were remained competent towards their job. These findings are similar to previous studies that revealed that more than half of respondents had high levels of emotional exhaustion and high levels of distrust (da Silva et al., 2014; Kathriarachchi & Rowntree, 2016). In addition, this study is also contrary to other studies that revealed that most respondents had reduced competence or personal accomplishments even though they had high levels of emotional exhaustion and depersonalization (Lasebikan and Oyetunde, 2012). The study agrees with study from Raftopoulos et al. (2012) portrayed that nurses find their job stressful.

Another study has also found out that intense and unsatisfactory work environment associated with long working hours and the suffering of work experiences in the intensive care unit were associated with burnout syndrome (Doulougeri et al., 2016).

### 5.1 Strategies of coping with burnout syndrome

Coping is a pattern of constructed behaviour towards situations deemed stressful, with the aim of avoiding its negative effects (Deklava et al., 2014). The outcome of this study is similar to a study that recommended strategies such as problem‐focused coping strategy, emotion‐focused strategy, avoidant coping strategy and humour for coping with burnout syndrome (Kathriarachchi & Rowntree, 2016). The document further added that some studies have also revealed that most popular coping strategies practised in stressful situations were listening to music, obtaining emotional support from family/friends and seeking comfort in religion.

## 6 CONCLUSIONS

Even though most nurses in this study feel emotionally exhausted from their job and have impersonal response towards recipients of the service, most assessed themselves as competent towards their job. Beside this, common causes of burnout among nurses include poor conditions of work, work overload, low wages, emotionally upsetting situations, handling a large number of patients alone, lack of break during shift and inadequate nursing staff, not being appreciated by in charges or nurse managers, inadequate resources to work with, bad attitude of other colleagues and nurse and manager conflict. Strategies that can be used to help resolve burnout or help nurses to cope with burnout in their various units include problem‐focused coping strategy, obtaining emotional support from family/friends strategy, using humour as a coping strategy, listening to music, emotion‐focused strategy, adequate motivation of staff, setting up hospital counselling units, providing adequate resources for nurses to work with, creating good working environment and good relationship with both staff and patients and increasing staff strength.

### 6.1 Strengths and weaknesses of the study

- The study might not be the first of its kind in Ghana but vividly spell out how HDU nurses feel about their work.
- It also showed that even though there were some existing coping strategies, some are not feasible, but respondents need to hold on to their job.
- The weakness was in the number of samples used—it was too small so generalization will not be possible.

### 6.2 Implication for nursing

The study set out to investigate among HDU nurses in a regional hospital what caused occupational burnout and how they cope with the stress. It was designed overtly with the intent of advocating for motivation of nurses for their hard work.

## CONFLICT OF INTEREST

The authors declare that they have no competing interests.

## AUTHOR CONTRIBUTIONS

YAB conceived the study, was the principal investigator and made most extensive contribution to the research. SO was involved in the conception of the research, guided the development of the proposal and supervised the study. IKA and AAD revised the research and developed a manuscript for its intellectual and professional content.

## ETHICAL APPROVAL

The study took an introductory letter from the school of Peri Operative, Korle‐Bu, Accra and a gatekeeper letter from research unit of the hospital. Voluntary participation was accorded with oral, written and signed consent.

## Data Availability

Data for this study will be available to any researcher upon reasonable request.
